# Surgical opportunities for primary prevention of high-grade serous ovarian cancer: a procedure-level analysis of opportunistic salpingectomy

**DOI:** 10.1186/s13048-026-02332-2

**Published:** 2026-11-07

**Authors:** Walid Shaalan, Christina Schmidt, Kyra Fischer, Friederike Stroisch, Felix K. F. Kommoss, Nourhan Hassan, Caroline Riedel, Helene Herrmann, Christina Koehler, Liat Matan, Cristina Taliento, Kathrin Hassdenteufel, Anna Sophie Scholz, Michael Samuel, Hannah Arndt, Andre Hennigs, Maximilian Riedel, Cecilie F. Torkildsen, Lisa Katharina Nees, Oliver Zivanovic, Fabian Riedel

**Affiliations:** 1 Department of Gynecology and Gynecological Oncology, University Hospital Heidelberg, Heidelberg Im Neuenheimer Feld 440, 69120 Heidelberg, Germany; 2 Institute of Pathology, University Hospital Heidelberg, Im Neuenheimer Feld 224, 69120 Heidelberg, Germany; 3 Center for Molecular Medicine Cologne, University of Cologne, Robert- Koch-Street 21, 50931 Cologne, Germany; 4 Biotechnology Department, Faculty of Science, Cairo University, Giza, 12613 Egypt; 5 Department of General Internal Medicine, Psychosomatics, and Psychotherapy, University Hospital Heidelberg, Im Neuenheimer Feld 410, 69120 Heidelberg, Germany; 6 Department of Obstetrics and Gynecology, School of Medicine and Health, Klinikum rechts der Isar, Technical University of Munich, Ismaninger Street 22, 81675 Munich, Germany; 7 Department of Clinical Science, Centre for Cancer Biomarkers CCBIO, University of Bergen, Bergen, 5020 Norway

**Keywords:** High-grade serous ovarian carcinoma, Opportunistic salpingectomy, Ovarian cancer prevention, Surgical opportunities, Procedure-level analysis

## Abstract

**Background:**

High-grade serous ovarian carcinoma (HGSOC), the most common and lethal subtype of epithelial ovarian cancer, is now recognized to originate predominantly from the distal fallopian tube. This paradigm shift has established opportunistic salpingectomy (OS) as a promising strategy for primary prevention during otherwise indicated abdominal or pelvic surgery. However, the frequency, timing, and surgical distribution of previous opportunities for OS among patients subsequently diagnosed with HGSOC remain insufficiently characterized.

**Objective:**

To quantify previous surgical opportunities for OS before HGSOC diagnosis using complementary patient-level and procedure-level analyses.

**Methods:**

This retrospective cohort study included patients with histologically confirmed HGSOC who underwent primary debulking surgery or interval debulking surgery after neoadjuvant chemotherapy between 2016 and 2025. Clinicopathological characteristics, family and personal cancer history, germline genetic testing results, and previous abdominopelvic procedures were reviewed. A procedure was considered eligible for OS if it was elective, had been performed at least 12 months before HGSOC diagnosis, and occurred after completion of childbearing.

**Results:**

Among 426 patients, 165 (38.7%) had undergone at least one previous abdominal or pelvic operation that fulfilled the predefined eligibility criteria for OS. At the procedure level, 210/469 previous operations (44.8%) represented potential opportunities for OS. Of these, 112/210 (53.3%) were sterilization procedures and 98/210 (46.7%) were non-sterilization procedures. Gynecologic surgery accounted for 135/210 (64.3%) eligible procedures, whereas 75/210 (35.7%) occurred during non-gynecologic surgery. Hysterectomy was the most common gynecologic procedure, and cholecystectomy was the most frequent non-gynecologic procedure. Eligible procedures were performed at a median age of 46.0 years (IQR, 40.0–53.0) and preceded HGSOC diagnosis by a mean interval of 17.9 years. Among 116 genetically tested patients meeting the OS-eligibility criteria, 32 (27.6%) carried a pathogenic germline variant.

**Conclusion:**

More than one-third of patients with HGSOC had at least one previous surgical opportunity during which OS could have been considered, nearly two decades before HGSOC diagnosis. Although gynecologic surgery remains the principal setting for implementing OS, more than one-third of eligible procedures occurred during non-gynecologic surgery. These findings highlight repeated opportunities for OS across surgical specialties and support broader multidisciplinary implementation of OS for the primary prevention of HGSOC.

**Supplementary Information:**

The online version contains supplementary material available at https://doi.org/10.1186/s13048-026-02332-2.

## Background

Ovarian cancer remains the most lethal gynecologic malignancy worldwide and continues to pose a major clinical and public health challenge because of its aggressive biological behavior, frequent diagnosis at an advanced stage, and limited effectiveness of screening strategies. In Germany, approximately 6,700 women are newly diagnosed with ovarian cancer annually, with more than two-thirds presenting with FIGO (International Federation of Gynecology and Obstetrics) stage III or IV disease at diagnosis [1]. Although outcomes have improved through advances in cytoreductive surgery, systemic therapy, and molecularly guided treatment, survival rates remain stage-dependent. Five-year overall survival exceeds 90% in patients diagnosed at an early stage but declines to approximately 40–45% in those presenting with advanced stage disease. These unfavourable survival outcomes highlight the need for effective primary prevention strategies [2].

Despite extensive efforts to improve early detection, no population-based strategy has demonstrated a reduction in ovarian cancer mortality. The UK Collaborative Trial of Ovarian Cancer Screening (UKCTOCS) revealed that screening with cancer antigen 125 and transvaginal ultrasonography did not significantly reduce ovarian-related and fallopian tube-related cancer deaths [3]. Consequently, increasing attention has shifted from early detection toward primary prevention, particularly for women at average population risk who account for the majority of ovarian cancer cases [4].

High-grade serous ovarian carcinoma (HGSOC) is the predominant histological subtype of epithelial ovarian cancer and accounts for the majority of ovarian-cancer related deaths. Over the past two decades, the understanding of HGSOC pathogenesis has changed fundamentally. Pathological examination of risk reducing salpingo-oophorectomy specimens from women with hereditary cancer susceptibility identified serous tubal intraepithelial carcinoma (STIC) and related precursor lesions within the distal fallopian tube [5]. Subsequent molecular and clinicopathological evidence established that a substantial proportion of HGSOCs originate from secretory epithelial cells of the fimbrial end of the fallopian tube rather than from the ovarian surface epithelium [6]. This paradigm shift provided the biological rationale for opportunistic salpingectomy (OS), which involves bilateral removal of the fallopian tubes during an indicated abdominal or pelvic procedure in women who have completed childbearing while preserving the ovaries. Rather than requiring a separate preventive operation, OS utilizes an existing surgical encounter to reduce future ovarian cancer risk [4, 7].

Following recognition of the fallopian tube as the principal site of origin for HGSOC, the Society of Gynecologic Oncology (SGO) published a clinical practice statement in 2013 recommending consideration of salpingectomy in women at average risk at the time of hysterectomy, in lieu of tubal ligation, and during other pelvic surgery [8]. Since then, several international societies, including the American College of Obstetricians and Gynecologists (ACOG), have issued similar recommendations, establishing OS as an increasingly accepted strategy for ovarian cancer prevention [9].

Population-based studies have demonstrated increasing implementation of OS, showing that its addition to hysterectomy or permanent sterilization requires only modest additional operative time without a clinically relevant increase in perioperative complications [10, 11]. Furthermore, a recent systematic review demonstrated that OS is associated with a substantial reduction in ovarian cancer risk, is cost-effective, and does not appear to adversely affect ovarian reserve or accelerate menopause [4]. Modeling studies further suggest that broad implementation of OS during eligible gynecologic and elective non-gynecologic abdominal procedures could substantially reduce the future incidence of HGSOC at the population level [12].

Opportunistic salpingectomy should, however, be distinguished from risk-reducing bilateral salpingo-oophorectomy, which remains the standard preventive intervention for women carrying pathogenic variants associated with hereditary ovarian cancer susceptibility [9].

Although implementation efforts have traditionally focused on gynecologic surgery, many women undergo elective non-gynecologic abdominal or pelvic procedures after completion of childbearing. Recent cost-effectiveness analyses suggest that incorporating OS into both gynecologic and selected non-gynecologic operations may further enhance the population-level impact of ovarian cancer prevention, supporting a multidisciplinary surgical strategy rather than an intervention restricted exclusively to gynecologic operations [12, 13].

Despite the increasing adoption of OS, important evidence gaps remain. Estimates of previous surgical opportunities vary considerably owing to differences in the definition of completed childbearing, the required interval between surgery and HGSOC diagnosis, and the criteria used to define eligible elective procedures. Furthermore, previous studies have primarily quantified surgical opportunities at the patient level, and little is known about the cumulative number and distribution of eligible procedures across a patient’s surgical history and across different surgical specialties [14].

The present study aimed to quantify previous surgical opportunities for OS among patients subsequently diagnosed with HGSOC using complementary patient-level and procedure-level analyses. By characterizing the number, timing, and surgical distribution of eligible procedures, this study further sought to inform future multidisciplinary implementation strategies for the primary prevention of HGSOC.

## Methods

### Study design and patient population

This retrospective cohort study included all patients with histologically confirmed HGSOC who underwent surgical management of newly diagnosed HGSOC at the Department of Gynecologic Oncology, Heidelberg University Hospital, between 2016 and 2025, including both primary debulking surgery and interval debulking surgery following neoadjuvant chemotherapy. Patients were eligible if HGSOC was confirmed by final histopathology and complete documentation of their previous surgical history was available. Patients with non-high-grade serous epithelial ovarian carcinoma, borderline ovarian tumors, primary peritoneal carcinoma, incomplete documentation of previous abdominal or pelvic surgical history, or a known pathogenic germline variant who had declined risk reducing surgery despite genetic counselling were excluded.

The study was conducted in accordance with the Declaration of Helsinki and was approved by the Institutional Review Board of Heidelberg University Hospital (IRB Approval Number: S-089/2025; March 10 2025).

### Recruitment and data collection

Demographic, clinicopathological, and operative data were extracted from the institutional electronic medical records and hospital information system, including age at HGSOC diagnosis, date of diagnosis, gravidity, parity, FIGO stage, family history of ovarian and/or breast cancer, personal history of malignancy, and germline genetic testing results.

A comprehensive review of each patient’s surgical history was performed to identify all documented abdominal and pelvic operations preceding the diagnosis of HGSOC. For each previous operation, patient age at surgery, year of surgery, surgical indication, surgical specialty, operative approach, and the interval between surgery and HGSOC diagnosis were recorded. Procedures were classified according to surgical specialty and categorized as gynecologic and non-gynecologic operations, with eligible procedures further classified as sterilization or non-sterilization procedures. Because patients could have undergone more than one previous operation, each procedure was evaluated independently, thereby enabling complementary patient-level and procedure-level analyses of potential opportunities for OS.

### Eligibility criteria for OS

A previous abdominal or pelvic operation was considered eligible for OS if all of the following criteria were fulfilled: [7, 14–17].

1. The operation was performed at least 12 months before HGSOC diagnosis.
2. The operation was performed after completion of childbearing, defined as women aged 45 years or older or following permanent sterilization irrespective of age.
3. The operation was an elective abdominal or pelvic procedure rather than an urgent or emergent intervention.

Surgical procedures performed after documented bilateral salpingectomy were not considered potential OS opportunities, as both fallopian tubes had already been removed.

### Statistical analysis

Statistical analyses were primarily descriptive, reflecting the exploratory nature of the study. Categorical variables were summarized using absolute frequencies and percentages, whereas continuous variables were reported as medians and interquartile ranges (IQRs). Data were analysed using IBM SPSS software version 20.0 (Armonk, NY, USA: IBM Corp.) and GraphPad Prism version 10.0 (GraphPad Software, Boston, MA, USA).

## Results

A total of 426 patients were identified during the study period. The median age at diagnosis was 64.0 years (IQR, 57.0–71.0). Nulligravidity was documented in 73/426 (17.1%) patients, whereas nulliparity was observed in 81/426 (19.0%) patients. A total of 385/426 (90.4%) patients presented with advanced-stage disease (FIGO III-IV). A family history of ovarian and/or breast cancer was reported in 162/426 (38.0%) patients. A personal history of malignancy was identified in 67/426 (15.7%) patients, with breast cancer being the most common previous malignancy (32/426, 7.5%). Germline genetic testing identified pathogenic variants in 98/426 (23.0%) patients. Baseline demographic and clinicopathological characteristics are summarized in Table 1.

Overall, 273/426 (64.1%) patients had undergone at least one abdominal or pelvic operation before the diagnosis of HGSOC. No documented cases of HGSOC following previous hysterectomy with bilateral salpingectomy were identified in the cohort (*n* = 0). Because some patients had undergone more than one previous operation, patient-level and procedure-level analyses are presented separately throughout the results. A total of 165/426 (38.7%) patients had at least one previous operation that fulfilled the predefined eligibility criteria for OS. No otherwise eligible procedures occurred between 6 and 12 months before HGSOC diagnosis. Among these patients, 129/165 (78.2%) had only one eligible procedure, whereas 36/165 (21.8%) experienced multiple opportunities for OS before HGSOC diagnosis, including two (28/165, 17.0%), three (7/165, 4.2%), or four (1/165, 0.6%) OS-eligible procedures (Table 1).

**Table 1:** Patient demographic and clinical characteristics (*N* = 426)

Age at diagnosis (years) Median (IQR)	64.0	57.0–71.0
**Gravidity**	**Number (N)**	**Percentage (%)**
No previous pregnancy	73	17.1
≥ 1 Pregnancy	340	79.8
Unknown	13	3.1
**Parity**		
Nulliparous	81	19.0
Primiparous	104	24.4
Multiparous	230	54.0
Unknown	11	2.6
**FIGO Stage**		
I + II	41	9.6
III + IV	385	90.4
**Family history of ovarian and/or breast cancer**		
No	264	62.0
Yes	162	38.0
**Personal history of malignancy**		
No	359	84.3
Yes	67	15.7
└─ Breast cancer	32	7.5
**Germline genetic testing**		
Pathogenic variant	98	23.0
Variant of uncertain significance (VUS)	10	2.3
Negative	200	46.9
Not performed/Unknown	118	27.7
**Prior abdominal or pelvic surgery**		
No	153	35.9
Yes	273	64.1
└─ Patients with **≥** 1 OS-eligible procedure	165	38.7*
└─ 1 OS-eligible procedure	129	78.2**
└─ 2 OS-eligible procedures	28	17.0**
└─ 3 OS-eligible procedures	7	4.2**
└─ 4 OS-eligible procedures	1	0.6**

Abbreviations: IQR, Interquartile range; FIGO, International Federation of Gynecology and Obstetrics; OS, opportunistic salpingectomy

*Percentage calculated from the whole cohort (*n* = 426)

**Percentage calculated among patients with ≥ 1 OS-eligible procedure (*n* = 165)

**Table 2:** A distribution of OS-eligible procedures according to surgical specialty (*N* = 210)

Surgical specialty	Number (*N*)	Percentage (%)
**Gynecology**	**135**	**64.3**
└─Hysterectomy	86	41.0
└─Tubal ligation	26	12.4
└─Unilateral salpingo-oophorectomy	9	4.3
└─Ovarian cystectomy	8	3.8
└─Myomectomy	5	2.4
└─Cesarean section	1	0.5
**General surgery**	**55**	**26.2**
└─Cholecystectomy	35	16.7
└─Hernia repair	10	4.8
└─Appendectomy	7	3.3
└─Small bowel resection	1	0.5
└─Splenectomy	1	0.5
└─Diagnostic laparoscopy	1	0.5
**Colorectal surgery**	**11**	**5.2**
└─Sigmoid resection	8	3.8
└─Hemicolectomy	3	1.4
**Hepato-pancreato-biliary surgery**	**3**	**1.4**
└─Pancreatectomy	2	1.0
└─Hepatectomy	1	0.5
**Urological surgery**	**4**	**1.9**
└─Nephrectomy	3	1.4
└─Adrenalectomy	1	0.5
**Transplant surgery**	**1**	**0.5**
└─Kidney transplant	1	0.5
**Bariatric surgery**	**1**	**0.5**
└─Sleeve gastrectomy	1	0.5

**Table 3:** Distribution of OS-eligible procedures according to indication (*N* = 210)

Procedure type	Number (*N*)	Percentage (%)
**Sterilization procedures**	**112**	**53.3**
└─Hysterectomy	86	41.0
└─Tubal ligation	26	12.4
**Non-sterilization procedures**	**98**	**46.7**
└─Gynecologic procedures	23	11.0
└─Non-gynecologic procedures	75	35.7

At the procedure level, 469 previous abdominopelvic operations were identified among the 273 patients with a history of previous surgery. Of these, 210/469 (44.8%) fulfilled the predefined eligibility criteria for OS. Across all surgical specialties, 135/210 (64.3%) OS-eligible procedures were performed within gynecologic surgery with hysterectomy representing the most common procedure (86/210, 41.0%). The remaining 75/210 (35.7%) non-gynecologic OS-eligible procedures were predominately cholecystectomies (35/210, 16.7%). Among the 35 cholecystectomies, 26/35 (74.3%) were performed laparoscopically and 9/35 (25.7%) via an open approach. In a sensitivity analysis excluding open cholecystectomies from OS eligibility, 201/469 (42.9%) procedures remained eligible, compared with 210/469 (44.8%) in the primary analysis.

General surgery accounted for 55/210 (26.2%) eligible procedures, followed by colorectal surgery (11/210, 5.2%), urological surgery (4/210, 1.9%), hepato-pancreato-biliary surgery (3/210, 1.4%), transplant surgery (1/210, 0.5%), and bariatric surgery (1/210, 0.5%).

Among the 165 patients with at least one OS-eligible surgical opportunity, the median ageat HGSOC diagnosis was 67.0 years (IQR, 60.0-74.0), whereas the median age at thecorresponding operation was 46.0 years (IQR, 40.0-53.0) (Table 4).

**Fig. 1:**
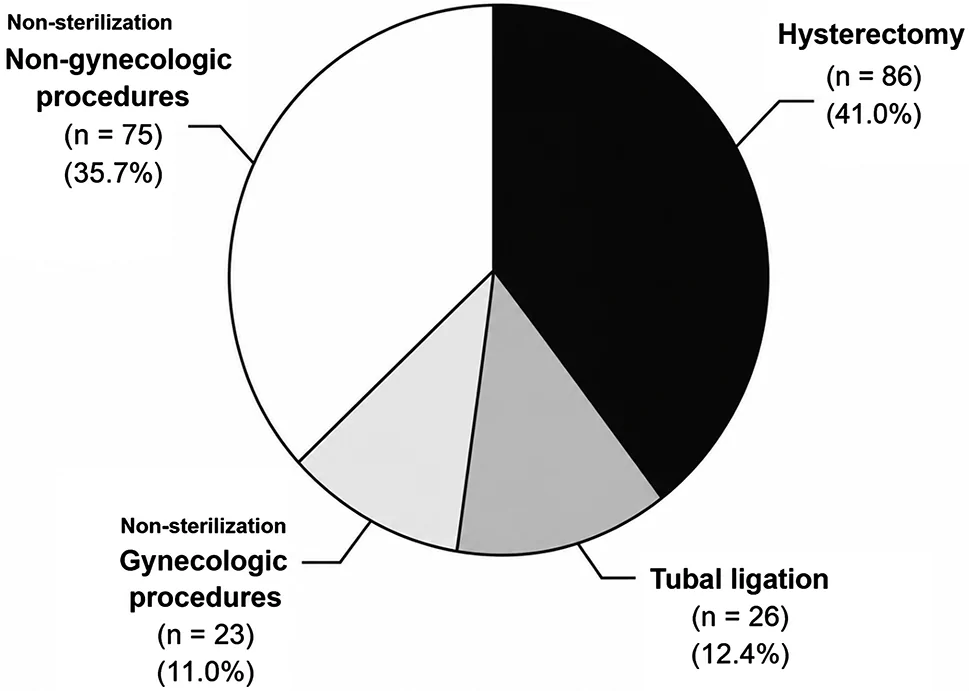
Distribution of OS-eligible procedures by surgical indication (*N* = 210)

Of the 210 OS-eligible procedures, 112/210 (53.3%) were hysterectomy or tubal ligation procedures, whereas 98/210 (46.7%) were other eligible abdominopelvic procedures, as summarized in Table 2 and 3 and Fig. 1.

**Fig. 2:**
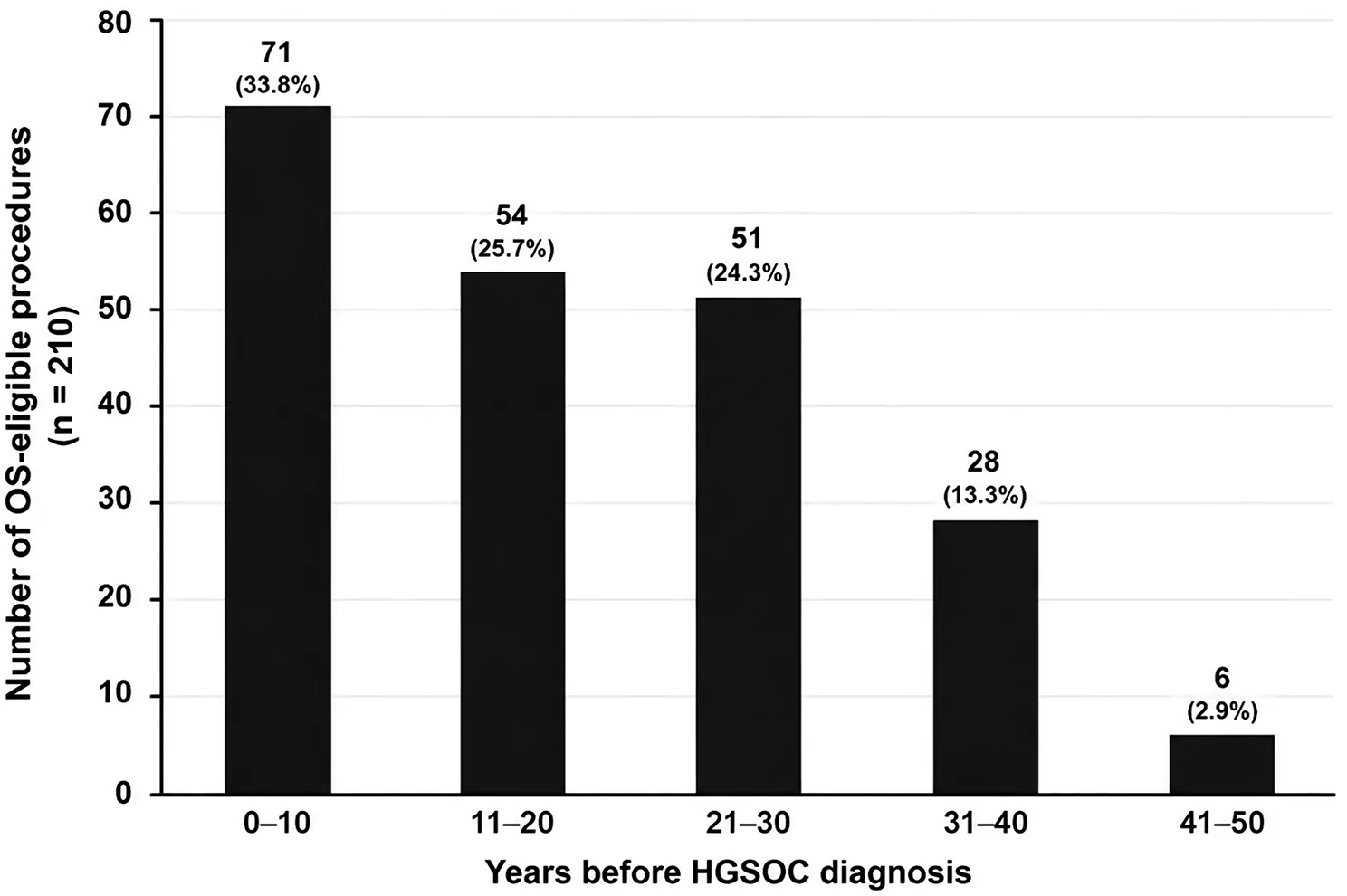
Time interval between OS-eligible procedures and HGSOC diagnosis (*N* = 210). Y- axis: Number of OS-eligible procedures. X- axis: Years before HGSOC diagnosis

Among the 210 OS-eligible procedures, 71/210 (33.8%) were performed 0–10 years before HGSOC diagnosis, 54/210 (25.7%) were performed 11–20 years before diagnosis, and 51/210 (24.3%) were performed 21–30 years before diagnosis. The remaining 34/210 (16.2%) procedures occurred more than 30 years before diagnosis. Overall, the identified surgical opportunities preceded HGSOC diagnosis by a mean interval of 17.9 years (Fig. 2).

**Fig. 3:**
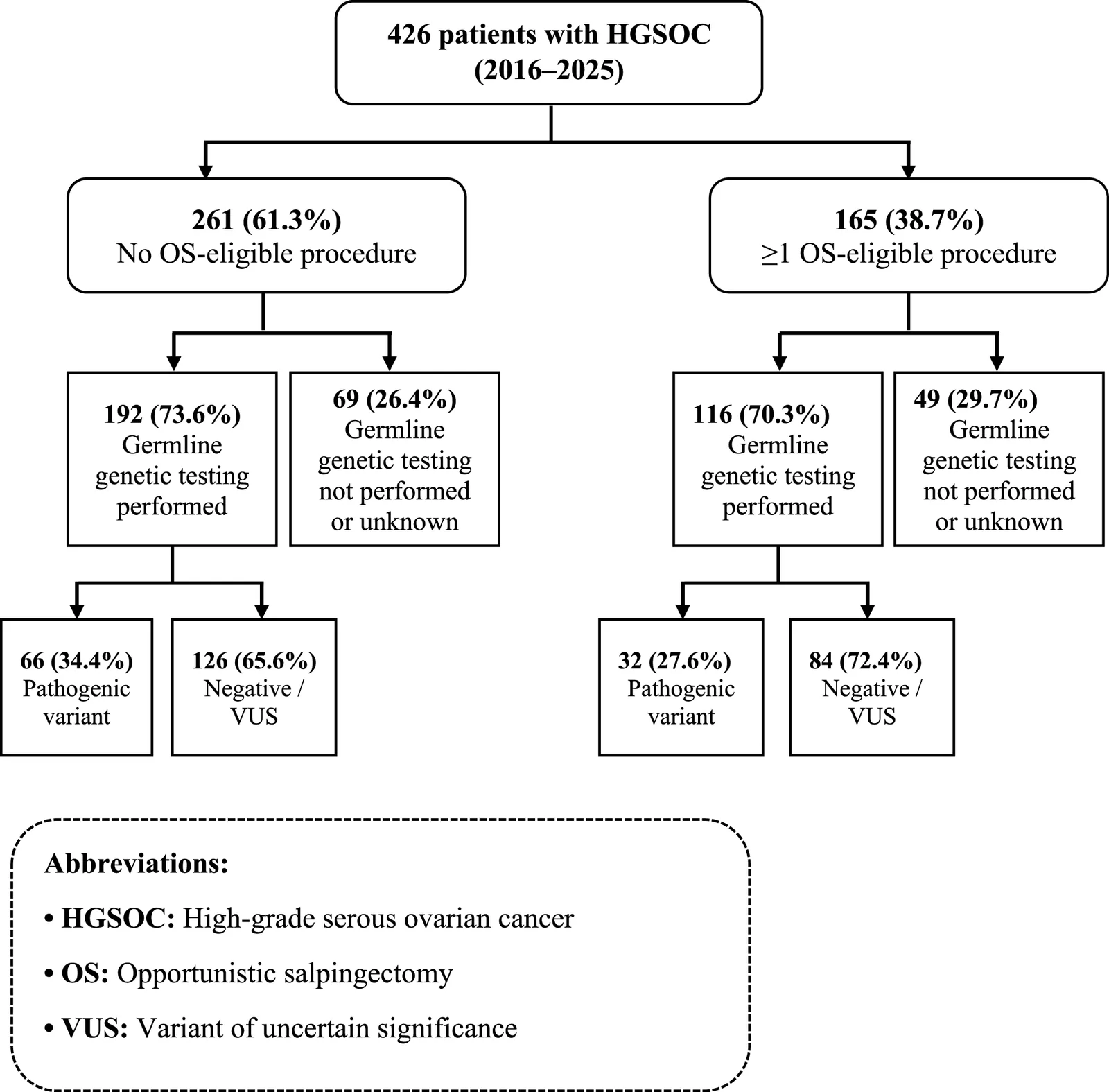
Flowchart of missed opportunities for opportunistic salpingectomy according to germline genetic testing

Across the entire cohort, 308/426 (72.3%) underwent germline genetic testing following HGSOC diagnosis, whereas 118/426 (27.7%) had no documented genetic testing. Among patients with at least one eligible opportunity for OS, 116/165 (70.3%) underwent testing, whereas 49/165 (29.7%) had no documented germline genetic testing. Among the 116 tested patients, 32/116 (27.6%) carried a pathogenic germline variant. The distribution of germline genetic testing results across the cohort is summarized in the flowchart (Fig. 3).

A subgroup analysis was performed among the 165 patients with at least one OS-eligible surgical opportunity. Of these, 66/165 (40.0%) had hereditary risk factors for ovarian cancer, defined by a personal history of breast cancer and/or a family history of ovarian and/or breast cancer in first- or second-degree relatives [18, 19].

## Discussion

The present single-center German study quantified previous surgical opportunities for OS in a contemporary cohort of patients diagnosed with HGSOC. Nearly two-thirds of patients had undergone at least one abdominopelvic operation before cancer diagnosis, and more than one-third had at least one procedure meeting the predefined eligibility criteria for OS. At the procedure level, almost half of all previous operations represented potential opportunities for OS. Although most eligible procedures were performed within gynecologic surgery, more than one-third occurred during non-gynecologic procedures, indicating that opportunities for ovarian cancer prevention extend beyond traditional gynecologic practice. Notably, eligible procedures preceded HGSOC diagnosis by a mean interval of nearly two decades, highlighting a substantial window of opportunity for primary prevention.

Over recent years, accumulating molecular and pathological evidence has established the distal fallopian tube as the principal site of origin for most HGSOCs, with STIC recognized as its precursor lesion, fundamentally shifting prevention strategies toward OS before malignant transformation [5, 20]. Consequently, OS has evolved from an optional adjunct during benign gynecologic surgery to an internationally endorsed cancer prevention strategy [9, 21]. Our findings demonstrate that a considerable proportion of women who developed HGSOC had missed opportunities for OS as a strategy for ovarian cancer prevention.

While previous studies have primarily quantified missed opportunities for OS at the patient level, our study additionally incorporated procedure-level analyses by evaluating every previous procedure individually. This approach provides a more comprehensive estimate of the cumulative preventive potential of OS across multiple surgical encounters. Interestingly, 36 of 165 patients (21.8%) experienced multiple eligible opportunities for OS before HGSOC diagnosis, highlighting the repeated nature of preventive opportunities throughout a patient’s surgical history. By capturing all eligible procedures rather than a single qualifying operation per patient, this procedure-level approach provides a more comprehensive assessment of cumulative surgical opportunities for OS across different surgical specialties before HGSOC diagnosis [14, 22].

In two recent retrospective studies by Moufarrij et al. and Tischer et al., reported opportunity rates for OS ranged from 23.7% to 56.5%, compared with 38.7% in our cohort [7, 14]. The heterogeneity in reported opportunity rates is potentially attributable to methodological differences between studies, including the criteria used to define previous surgical opportunities and the classification of eligible procedures, rather than true differences in the prevalence of surgical opportunities. These findings highlight the need for standardized eligibility criteria to more accurately estimate the population-level preventive potential of OS.

Practice patterns of OS have evolved considerably over the past decade. In Germany, salpingectomy has become increasingly established in routine gynecologic practice. National data demonstrated an approximately fourfold increase in salpingectomies between 2005 and 2020, from 12,286 to 50,398 procedures. By 2020, 45% of hysterectomies were combined with salpingectomy, exceeding 65% among women aged 35–49 years. Furthermore, the proportion of surveyed German gynecologists performing OS in > 50% or all applicable cases increased from 56.6% in 2015 to 89.0% in 2022. These findings demonstrate the increasing integration of OS into routine gynecologic practice in Germany [23]. Beyond hysterectomy, salpingectomy is increasingly considered as an alternative to tubal ligation for permanent contraception, including at the time of Caesarean delivery, with available evidence supporting its feasibility and safety in selected patients [9, 21].

Our findings indicate that opportunities for ovarian cancer prevention extend beyond gynecologic surgery. Although hysterectomy was the most frequent gynecologic operation, cholecystectomy represented the predominant non-gynecologic opportunity. This suggests that incorporating OS into elective non-gynecologic procedures may broaden its population-level preventive impact. Supporting this concept, Tomasch et al. demonstrated that OS could be successfully performed during elective laparoscopic cholecystectomy in more than 90% of patients, with only a modest increase in operative time and no significant salpingectomy-related complications [24].

While prospective evidence supports the feasibility of OS during elective laparoscopic cholecystectomy [24], pelvic access during open upper-abdominal procedures may be limited. Similarly, during complex procedures such as bariatric or transplant surgery, the feasibility of OS should be assessed individually according to the operative approach, pelvic accessibility, and potential surgical complexity. Excluding open cholecystectomies resulted in only a modest reduction in the proportion of potential OS opportunities (44.8% vs. 42.9%), suggesting that the overall procedure-level findings were not substantially affected. Nevertheless, these estimates represent potential preventive surgical encounters and do not imply that OS should be routinely performed during all identified procedures, irrespective of the technical feasibility.

The feasibility of a broader multidisciplinary approach is further supported by accumulating evidence. In a systematic review of 158 studies, Kahn et al. reported that OS may reduce ovarian cancer risk by up to 80% without increasing perioperative morbidity or adversely affecting ovarian function [4]. Consistent with these findings, long-term population-based data from Hanley et al. and prospective evidence from Venturella et al. showed that OS can be implemented safely without increased surgical complications or the risk of premature menopause [25, 26]. Accordingly, the principal challenge is no longer to establish the safety and feasibility of OS, but to develop effective implementation strategies across surgical specialties and healthcare systems.

Previous implementation studies have identified limited awareness among non-gynecologic surgeons, inconsistent patient counselling and institutional barriers as major obstacles to broader adoption. Gilgannon et al. emphasized that extending OS into general surgery requires dedicated multidisciplinary pathways addressing patient selection, informed consent, operative workflow, and postoperative documentation, rather than simply introducing an additional surgical technique [27]. Similarly, Runnebaum et al. highlighted that OS should be regarded as a shared preventive responsibility among surgeons performing elective abdominal procedures in women who have completed childbearing [23, 28].

The prolonged interval between previous surgery and cancer diagnosis, nearly two decades on average in our cohort, further emphasizes the potential for primary prevention. This latency is consistent with the current understanding of tubal carcinogenesis and underscores that elective abdominal surgery after completion of childbearing may represent a unique opportunity to interrupt this carcinogenic process [5, 25].

Furthermore, our results demonstrated that nearly one-third (27.6%) of patients with an OS-eligible opportunity who underwent germline testing carried a pathogenic cancer-predisposition variant. As testing was performed only after HGSOC diagnosis, hereditary susceptibility remained unrecognized at the time of the eligible procedure, potentially representing a missed opportunity for individualized ovarian cancer prevention [29–31].

Notably, 40% of patients eligible for OS had hereditary risk factors for ovarian cancer. This observation supports further evaluation of integrated preoperative pathways that combine counselling for OS with assessment of hereditary cancer susceptibility, enabling a more preventive approach [19, 31].

The procedure-level analysis indicates that opportunities for ovarian cancer prevention may recur throughout a patient’s surgical history and across different surgical specialties. Accordingly, OS may be considered whenever the fallopian tubes are safely accessible during elective abdominal or pelvic surgery after completion of childbearing and can be performed without increasing the complexity or risk of the primary procedure, rather than being restricted to hysterectomy or sterilization [27, 28].

Translating this concept into routine practice will require multidisciplinary implementation strategies addressing patient selection, preoperative counselling, operative coordination, institutional barriers, and structured education across surgical specialties to facilitate broader adoption of OS [32, 33].

A major strength of this study is the procedure-level evaluation of previous abdominal and pelvic operations, enabling complementary patient-level and procedure-level analyses of potential opportunities for OS. Detailed operative documentation and assessment of hereditary-risk features further enhance the robustness and clinical relevance of the findings. However, potential limitations should be considered. The single-center design may limit the generalizability of these findings. Eligibility for OS relied on retrospective assumptions regarding completion of childbearing, and many included procedures preceded the current understanding of tubal carcinogenesis and recommendations supporting OS. Furthermore, the retrospective classification of non-gynecologic procedures as potential OS opportunities did not allow case-specific assessment of technical feasibility.

Future studies should assess standardized implementation pathways, technical feasibility, patient preference, and long-term oncologic outcomes to determine the population-level impact of OS as an ovarian cancer preventive strategy.

## Conclusion

More than one-third of patients with HGSOC had at least one previous surgical opportunity during which OS could have been considered, often nearly two decades before diagnosis. Although gynecologic surgery remains the principal setting for implementation, a substantial proportion of opportunities occurred during non-gynecologic abdominal procedures, highlighting the potential to expand ovarian cancer prevention beyond traditional gynecologic practice. The procedure-level approach adopted in this study provides a comprehensive framework for identifying potential preventive opportunities across surgical specialties and supports further evaluation of OS in appropriately selected multidisciplinary surgical settings as a strategy for the primary prevention of HGSOC.

**Table 4:** Age at OS-eligible surgery and HGSOC diagnosis (*n* = 165)

Age in years	Minimum - Maximum	Median	IQR
Age at HGSOC Diagnosis	44.0–86.0	67.0	60.0–74.0
Age at OS-eligible Surgery	21.0–77.0	46.0	40.0–53.0

Abbreviations: HGSOC, High-grade serous ovarian carcinoma

## Supplementary Information

Below is the link to the electronic supplementary material.

Supplementary Material 1

## Data Availability

All data generated or analyzed during this study are contained within the article. Further enquiries can be directed to the corresponding author.

## Abbreviations

ACOG: American College of Obstetricians and Gynecologists
FIGO: International Federation of Gynecology and Obstetrics
HGSOC: High-grade serous ovarian carcinoma
IQRs: Interquartile ranges
IRB: Institutional Review Board
OS: Opportunistic salpingectomy
SGO: Society of Gynecologic Oncology
STIC: Serous tubal intraepithelial carcinoma
UKCTOCS: UK Collaborative Trial of Ovarian Cancer Screening
